# Prodromes and Preclinical Detection of Brain Diseases: Surveying the Ethical Landscape of Predicting Brain Health

**DOI:** 10.1523/ENEURO.0439-18.2019

**Published:** 2019-07-25

**Authors:** Nathan S. Ahlgrim, Kristie Garza, Carlie Hoffman, Karen S. Rommelfanger

**Affiliations:** 1 Graduate Program in Neuroscience, Emory University, Atlanta 30322, GA; 2 Department of Neurology, Emory University, Atlanta 30322, GA; 3 Department of Psychiatry and Behavioral Sciences, Emory University, Atlanta 30322, GA; 4 Center for Ethics, Neuroethics Program, Emory University, Atlanta 30322, GA

**Keywords:** Alzheimer, autism, neuroethics, preclinical detection, prodrome, schizophrenia

## Abstract

The future of medicine lies not primarily in cures but in disease modification and prevention. While the science of preclinical detection is young, it is moving rapidly. Preclinical interventions offer hope to decrease the severity of a disease or delay the development of a disorder. With such promise, the research and practice of detecting brain disorders at a preclinical stage present unique ethical challenges that must be addressed to ensure the benefit of these technologies. Direct brain interventions have the potential to impact not just what a patient has but who they are and who they could become. Further, receiving an assessment for a preclinical or prodromal state has potential to impact perceptions about capacity, autonomy and personhood and could become entangled with stigma and discrimination. Exploring ethical issues alongside and integrated into the experimental design and research of these technologies is critical. This review will highlight ethical issues attendant to the current and near future states of preclinical detection across the life span, specifically as it relates to autism spectrum disorder (ASD), schizophrenia, and Alzheimer’s disease.

## Significance Statement

Preclinical interventions offer the strongest promise of delaying, modifying, or preventing the development of clinical brain disorders. Although promising, intervening at early stages in disorders inherently linked to identity and personhood presents unique ethical challenges. These challenges must be addressed before the practices are implemented. Both the treatment and the diagnosis have the potential to profoundly impact patients. We contextualize the risk of diagnosing preclinical states and present the limitations of preclinical interventions to guide research and policy as the field of preclinical detection rapidly expands.

## Introduction

Early intervention and disease modification are the future of health care worldwide. Rather than the technical and regulatory concerns, the greatest threat to this effort of detecting prodromal and preclinical states may in fact be ethical issues. Detecting diseases and disorders before clinical symptoms manifest enables earlier intervention and offers the hope of improved health outcomes. For example, screening for markers of breast cancer before symptoms arise is both widespread and recommended by many physician groups (Monticciolo et al., 2017; Sardanelli et al., 2017). Early-stage interventions reduce average patient cost by more than $100,000 over two years (Blumen et al., 2016) and decrease mortality (Howlader et al., 2017). Such a large positive effect of early detection and treatment provide an almost incontrovertible argument for regular early screenings. Even so, the method of arriving at early intervention is controversial. There is conflicting evidence on the efficacy of routine mammograms in decreasing breast cancer mortality (Berry et al., 2005; Domchek et al., 2010; Narod et al., 2014; Harding et al., 2015; Monticciolo et al., 2017). Whether regular screenings for breast cancer are necessary is an ongoing debate, demonstrating the complexities that arise from early detection efforts, even when treatments are widely available and effective. The debate becomes more complicated with disorders where effective treatments are not yet developed, as with brain disorders.

With the considerable global burden of brain disease, the promise of early detection and early intervention cannot be overstated. That being said, preclinical detection of brain disorders encompasses a unique suite of ethical concerns, as dysfunctions in the brain directly impact behavior and are intrinsically linked to identity and autonomy. In other words, when we predict a future brain disorder, we not only predict a health diagnosis but also predict who a person may become.

This review will discuss the considerations surrounding the ethics of preclinical detection through the lens of three brain disorders that typically present at distinct time points across the life span: autism spectrum disorder (ASD) in early childhood, schizophrenia in adolescence, and Alzheimer’s disease with aging populations. A patient is similarly impacted whether the etiology of a disorder is an acute biological or a multifactorial biopsychosocial one, so disorders from both categories will be discussed together. Related discussions of the ethics of preclinical detection have been started in other venues, such as Baum (2016) and Chneiweiss (2017). We will expand the discussion and place a greater emphasis on the implications for patients in a medicalized preclinical state. The disorders we focus on demonstrate the unique ethical quandaries in: (1) risk/benefit analysis, (2) the possibility of stigma and discrimination, and (3) responsibility and communication of risk. The review will conclude with recommendations for addressing these ethical challenges, which we do not intend to hinder research but to anticipate and mitigate potential roadblocks. As medical screenings and diagnostic tools continue to expand in scope and accuracy, an ethical framework will be necessary, even in research and clinical settings where preclinical detection of brain disorders is not the primary goal. The nature of preclinical detection is inherently probabilistic, so certainty can never be fully achieved with these strategies, but citizens worldwide stand to greatly benefit from the scientific advancements offered by preclinical detection if interventions and regulation are developed with careful ethical reflection. We believe addressing these ethical concerns in anticipation and as part of the improvements to preclinical detection technology will help ensure the promise of improved health that predictive technologies aspire to offer.

## Terminology: Preclinical or Prodromal Brain Disorders

Brain disorders are contextualized states, regardless of their etiology. Disordered states that lead to disordered behavior are diverse in their development and manifestation, and some of these states are not universally seen as truly disordered (e.g., the prominent neurodiversity movement in the ASD community; Armstrong, 2015). That said, all cases discussed here, and all cases in which preclinical detection could be used to identify patients before symptom onset, are medicalized, and are therefore subject to the same protection, concerns, and risks. The preclinical label is defined by the presence of predictive markers in the absence of symptoms that currently define the disease. Preclinical states are distinct from prodromal or subclinical states, in which some clinical presentations (such as a mood disorder) are present but do not satisfy criteria for diagnosing a disorder (like schizophrenia; Gourzis et al., 2002; Meyer et al., 2005; for examples of preclinical and prodromal markers, see Table 1, adapted from Arias et al., 2018). Early interventions of schizophrenia currently target the prodromal stage. In ASD, the hope is that early interventions begin at the age when the child’s behavioral symptoms do not yet reach diagnostic criteria. Efforts in Alzheimer’s disease are unique, in that the preclinical stage has been defined by an absence of behavioral or cognitive symptoms, well before the onset of mild cognitive impairment (MCI). The definition and detection of preclinical stages are more accessible in disorders like Alzheimer’s disease, which have established molecular biomarkers (e.g., measuring amyloid levels with positron emission tomography and measuring τ levels in cerebral spinal fluid (Dubois et al., 2016; see Table 1) arising well before behavioral symptoms. Preclinical Alzheimer’s disease is defined as the presence of one or more of these molecular biomarkers, in the absence of cognitive impairment. The diagnosis is often subdivided into two differential diagnoses: presymptomatic, for those who will develop clinical Alzheimer’s disease with pathogenic autosomal mutations, and asymptomatic, for those at risk of developing clinical Alzheimer’s disease with predictive biomarkers (Dubois et al., 2010). The reliability and validity of such tests will be further explored in the following section. In contrast to Alzheimer’s disease, no preclinical biomarkers for ASD or schizophrenia have been validated to date, although many genetic and environmental factors have been identified. Current efforts for early detection in these diseases focus on identifying subclinical symptoms in the prodrome (Gourzis et al., 2002; Christensen et al., 2016)

**Table 1.** Recognized biomarkers, symptoms, and methods for detection

	Preclinical biomarkers	Prodromal symptoms	Techniques for measuring markers or symptoms
Autism	None identified	Decreased social engagement and eye focus (Jones and Klin, 2013)	Eye tracking (Klin et al., 2002), naturalistic observation (Baranek, 1999), structural brain scan (Hazlett et al., 2017)
Schizophrenia	None identified	Subclinical positive, negative, and cognitive symptoms (Goulding et al., 2013)	Clinical interview (Goulding et al., 2013), genomic analysis (Schizophrenia Working Group of the Psychiatric Genomics Consortium, 2014)
Alzheimer’s	Low CSF Aβ_1-42_ with high CSF P-τ or T-τ, increased amyloid PET retention, autosomal dominant mutation (e.g., APP, PSEN1/2; Jack et al., 2011; Dubois et al., 2014, 2016)	Mild cognitive impairment	PET scan with injectable tracer, lumbar puncture, memory assessment (e.g., FCSRT; Dubois et al., 2016)

Alzheimer’s is the only disease of those discussed with recognized preclinical markers. Adapted from Arias et al. (2018). CSF: cerebrospinal fluid, PET: positron emission tomography, FCSRT: Free and Cued Selective Reminding Test, APP: amyloid protein precursor, PSEN: presenilin.

## Current Science and Assessment Techniques in Preclinical Detection

Detection and assessment techniques for preclinical brain disorders are currently restricted to research efforts (including clinical trials); none are implemented in routine clinical practice. Even so, the use of “big data” medicine (e.g., whole-genome sequencing) expands the opportunity for preclinical detection to occur as a secondary outcome of an unrelated test or procedure. That said, the utility of early interventions is pushing clinicians to incorporate screening practices for early stages of disease.

Parents were historically the initiators of an eventual ASD diagnosis, but efforts to increase awareness and validate screening protocols have shifted the responsibility to clinicians. Although governmental recommendations do not support population screening procedures, many advising committees say otherwise (Committee on Children with Disabilities, 2001; Zwaigenbaum et al., 2015; American Academy of Pediatrics, 2017). As a result, a growing number of clinicians have adopted routine screenings as a part of their practice (Palmer et al., 2011; Coury et al., 2017). Similarly, clinicians are now also recommended to screen older adults for early signs of dementia (McKhann et al., 2011; Cordell et al., 2013), and the cost for such screenings are covered by the American Medicare system. Recommendations for including biomarker screening for Alzheimer’s disease is pending further validation of the methods. In contrast, there are no commonly implemented screenings for the development of schizophrenia before help-seeking is initiated by the patient or caregiver (Larson et al., 2010; Seidman et al., 2010).

Below, we will provide an overview of the state of preclinical and prodromal detection throughout the lifespan. Complementary, if somewhat separate, opportunities for early detection exist in the realm of digital phenotyping and incidental findings. Digital phenotyping relies on passive data collection from smartphone and other technology to predict the development of brain and mental health disorders (Jain et al., 2015; Torous et al., 2016). Incidental findings refer to clinically relevant results that were not the primary purpose of a diagnostic test. A significant body of scholarship has addressed how and whether to ethically disclose incidental findings, taking the perspectives of many stakeholders into account (Illes et al., 2004; Haga et al., 2012a,b; Wolf et al., 2012; Kleiderman et al., 2014). The ethical guidelines for incidental findings can serve as a model for how to incorporate preclinical detection, but new frameworks will be required. An incidental finding of a preclinical brain disorder has different social and personal implications than that of other diseases and must be handled accordingly. Here, we will focus on the development and implementation of biomarkers for brain disorders. While we focus on ASD, schizophrenia, and Alzheimer’s disease due to their prevalence and the significant amount of ongoing research in those fields, similar biomarker research exists for other disorders, such as multiple sclerosis, Parkinson’s disease, Lewy body dementia. The rapid development of detection measures, pressure to implement them in clinical practice, and the ethical issues attendant, even during the research phase, warrant immediate discussion.

### ASD

ASD encompasses a range of phenotypes, from mild social impairment to an inability for self-sufficiency (American Psychiatric Association, 2013). ASD is now estimated to affect one in 160 children globally (World Health Organization, 2017) and is the leading cause of disability in children under the age of five (Baxter et al., 2015). The average age of diagnosis is approximately four years old (Christensen et al., 2016), which makes the needs of patients and their caregiver(s) a public health concern (Khanna et al., 2011; Cadman et al., 2012).

Studies have shown that infants who will develop autism prefer looking at mouths versus eyes during social engagement (Jones and Klin, 2013). Early screening attempts for ASD rely on eye-tracking in infants to detect atypical patterns of social gaze. Retrospective analyses of eye tracking behavior have identified infants as young as six months of age who would later develop ASD (Chawarska et al., 2013; Jones and Klin, 2013; Shic et al., 2014). To date, these studies test the value of eye-tracking as a relatively non-invasive, easy, and inexpensive screening tool. These studies target high-risk populations (siblings of children with autism) of infants and children whose parents express concern over their child’s social development (Sandin et al., 2014; Rowberry et al., 2015). Eventually, the hope is that such a tool could be implemented in routine wellness visits in all infants (high risk or not). Preliminary studies have also found differences in cortical development between infants who do and do not develop ASD (Hazlett et al., 2017). While brain scans may provide an opportunity for another preclinical biomarker of the disorder, neuroimaging is likely less accessible and too expensive to be considered for widespread screening. Early interventions to address early diagnoses are currently being designed. Perhaps unique to ASD treatment, the proposed behavioral interventions are beneficial for both autistic children and typically developing children (Institutes of Medicine and National Research Council, 2013), which minimizes the risk of false positives in this specific context.

### Schizophrenia

Schizophrenia develops later in life than ASD, with the first symptoms usually appearing in late adolescence/early adulthood or during the peri-menopausal phase (Castle and Murray, 1993; World Health Organization, 2001). Positive symptoms (such as psychosis), negative symptoms (such as anhedonia), and cognitive deficits contribute to the severe disability and loss of productivity associated with the disorder (World Health Organization, 2001). Although the lifetime prevalence of schizophrenia is ∼1% of the world population, the World Health Organization (WHO) estimates that schizophrenia is the eighth leading cause of disability-adjusted life years (DALYs) in 15–44 year olds (World Health Organization, 2001). Many risk factors of schizophrenia have been identified, including environmental (Cornblatt et al., 2003) and genetic (Schizophrenia Working Group of the Psychiatric Genomics Consortium, 2014) contributors. Despite the genetic factors, genome-wide association studies (GWAS) show low sensitivity and specificity in identifying those who will develop schizophrenia, which has led some teams to warn against using genetic analyses as predictive tests (Schizophrenia Working Group of the Psychiatric Genomics Consortium, 2014). No preclinical markers of schizophrenia have been identified; as such, clinicians rely on prodromal symptoms like anxiety, sleep disturbances, and depressive mood, to identify at-risk patients (Goulding et al., 2013).

At-risk patients are often identified because of treatment sought by the patient or caregiver, not by routine appointments. People often seek treatment for prodromal symptoms for schizophrenia, which are themselves clinical symptoms for other disorders (Gourzis et al., 2002; Meyer et al., 2005; Rosen et al., 2006). At this early stage, symptoms, family history, and genetic risk factors can put the patient at a high-risk for developing schizophrenia (Larson et al., 2010; Seidman et al., 2010; Goulding et al., 2013). This categorization presents the opportunity to intervene before clinical schizophrenia develops, in the interest of instigating preventative interventions. Prodromal symptoms do not always transition into clinical schizophrenia. Symptoms are often non-specific to psychosis (Gourzis et al., 2002; Rosen et al., 2006), and this has hindered success in designing early interventions. Prodromal interventions, such as the use of atypical antipsychotics (McGorry et al., 2009), antidepressants (Cornblatt et al., 2007), and alternative treatments like omega-3 fatty acids (Amminger et al., 2010), have produced mixed success in reducing transition rates (Larson et al., 2010). The uncertainty of a prodromal diagnosis further limits the confidence of successfully intervening before clinical symptoms develop, especially given the severity of side effects of anti-psychotic medications (Patel et al., 2014).

### Alzheimer’s disease

Alzheimer’s disease is unique among the three disorders discussed here, in that there is a generally accepted symptomatic subclinical stage for this disorder (MCI), which is often preceded by the presence of amyloid-β (Aβ) plaques, tau, and neurodegenerative biomarkers (Dubois et al., 2014; Jack et al., 2016; Racine et al., 2017). The research has progressed to the point that many organizations are advocating for the inclusion of a preclinical (fully asymptomatic) diagnosis being integrated into regular clinical practice (Dubois et al., 2014; Alzheimer’s Association, 2019). Alzheimer’s disease is the leading cause of dementia, and risk for this disorder increases dramatically with age (Hebert et al., 2013). Occurrence of the disorder is expected to double in the next 20 years, driven largely by the impending boom in population of those aged 65 or older (He et al., 2016). Ranked as the 25th most burdensome disorder in 1990, the increasing prevalence has driven Alzheimer’s disease to become the 12th most burdensome disorder in the United States over the past 20 years (Alzheimer’s Association, 2019). Similar increases in prevalence and burden are recorded throughout Europe (Wittchen et al., 2011). The protracted development of the disorder creates an enormous burden on the primary caregiver(s), as many as 40% of whom suffer from depression (Alzheimer’s Association, 2019).

In recent years, preclinical trials have commanded more of the industry’s effort, given the poor success rate of pharmaceutical trials in clinical interventions (Cummings et al., 2014; Hung and Fu, 2017). Dementia is thought to develop 20–30 years after the onset of Aβ deposits in the brain (Hubbard et al., 1990; Jansen et al., 2015), strongly supporting the idea that effective treatments may require intervening at the preclinical stage. Multiple ongoing clinical trials for pharmaceutical interventions now target high-risk populations not yet diagnosed with any cognitive impairment. For example, many drugs that previously failed efficacy trials in patients with mild to moderate Alzheimer’s disease are now being retested in preclinical populations (Hung and Fu, 2017). High-risk populations are defined as individuals with a family history of Alzheimer’s disease (Honea et al., 2012), the ε4 allele of the APOE gene (Bonham et al., 2016), or the presence of biomarkers, like elevated τ and a high Aβ_1-42_/Aβ_1-40_ ratio (Holland et al., 2012).

## Balancing Risks and Benefits

### Patient protection

Participants for trials of preclinical detection and/or treatment are most often recruited from “high-risk” populations, e.g., a family history of ASD or Alzheimer’s disease, or a diagnosis of prodromal schizophrenia. Researchers and clinicians involved in these studies must therefore make conscious efforts to minimize the risk of coercion and to discourage unsubstantiated hopes that the research will personally benefit the participants, known as therapeutic misconception (Appelbaum et al., 1982). Research participants given a hypothetical high-risk status for Alzheimer’s disease cited the desire to lower personal risk of developing dementia as a reason for enrolling in preclinical research more often than subjects given a normal risk status. The discrepancy between the groups remained even when informed that the efficacy of preclinical interventions has not been established (Grill et al., 2013). This evidence demonstrates that high-risk populations are inherently vulnerable to have their judgment clouded by the promises of preclinical detection, and thus their autonomy and consent must be carefully addressed. The need to protect against therapeutic misconception is perhaps the most widely discussed protection for patients, but the research community also stands to benefit from clarifying therapeutic misconceptions. “Research tourism,” or the practice of enrolling in studies for the express purpose of obtaining diagnoses or treatments (Townsend and Cox, 2013; Gibson et al., 2017), certainly demonstrate the challenge of therapeutic misconception of many clinically oriented scientific efforts. However, enrolling such patients could jeopardize the validity of the studies, since patients motivated by research tourism are likely to carry high-risk factors or be in the early stages of a disorder.

Any personal benefits that could be gained from preclinical detection are dependent on current and future research in the science of therapeutic interventions. Reducing lifetime cost and minimizing suffering by intervening early are possible via preclinical detection. However, these outcomes are not guaranteed in ASD, schizophrenia, Alzheimer’s disease, or any other condition being explored for preclinical and prodromal markers. Evidence suggests that early interventions like applied behavioral analysis (ABA) and antipsychotic treatment improve outcomes in ASD (Estes et al., 2015) and schizophrenia (McGorry et al., 2002; Woods et al., 2003; Kulhara et al., 2008), respectively. Even so, the positive effects of preclinical intervention are difficult to quantify. At best, successful interventions prevent the progression to clinical disease. Since all preclinical states are defined by a risk of progressing to the clinical disorder, large studies are required to statistically differentiate between patients who were successfully treated and those who would not have developed the disorder with or without treatment.

Given the early stages of this research, the limited personal benefits available to the patients must be emphasized by the research staff in the consent process to ensure fully informed consent. Participating in research for personal health benefit is not unethical, but it is unethical for the research team to falsely inflate the benefits to incentivize participation. Even in the absence of overpromising, the public are active consumers of an optimistic and hyped media that offers its own priming for hope. Therefore, ongoing updates with multiple stakeholders and public scholarship must be integral to the research process.

### Communication of information

Another challenge of communication happens during the research process wherein researchers face the dilemma of when and how much information should be communicated to the research participant. Decisions on whether to disclose preclinical status, considering its impact on identity and autonomy, must be considered with a deep knowledge of the specific population being served. Although some patients may appreciate the opportunity to plan for a developing disorder, others may find the diagnosis more distressing than helpful. When presented with the opportunity to participate in a hypothetical preclinical Alzheimer’s disease study, participants were as likely to enroll whether or not they would be informed of their amyloid status (Grill et al., 2016). Still, the psychological effects of being given such information should not be assumed to be as inconsequential as the choice to receive it. Recognizing the potential for distress, the International Working Group (IWG) has recommended that doctors not disclose preclinical Alzheimer’s disease status by default, but only “when well-informed subjects request the information, in cases of high level of social responsibility and cognitive demand or in cases of inclusion in research protocols and clinical trials” (Dubois et al., 2016). This is the primary difference between a disclosure of a preclinical diagnosis and an incidental finding in research efforts like brain imaging or whole-genome sequencing. Many argue that it is unethical to withhold incidental findings when the finding would trigger a specific course of action and treatment (Chneiweiss, 2017). That argument is not applicable to a preclinical state because there are currently no proven courses of action to treat a preclinical state. Therefore, the decision of whether to disclose a preclinical state to a patient must be a part of the consent process, and the choice should not dictate a patient’s participation in the study or trial. Such recommendations only address the choice of participants knowing their status; more protections will be necessary once the screening technology expands beyond the research sector and into commercial opportunities.

Many clinicians hope that disclosing high-risk or preclinical status will promote health-positive behaviors in patients hoping to mitigate the progression of the disease. Indeed, disclosure of risk status (by APOE4 genotype, a risk factor for Alzheimer’s disease) significantly increases Alzheimer’s-specific health-positive behavior changes, even when participants are specifically informed that no preventative behaviors are empirically supported (Chao et al., 2008). Further, a preclinical diagnosis for diseases that have no effective treatments, as in Alzheimer’s disease, may increase the monitoring of symptoms. Diligent monitoring and screenings could enable earlier intervention once clinical symptoms develop. Decades of data following breast cancer screenings have demonstrated that women tend to increase their vigilance following a positive BRCA1 test, with increased mammogram screenings (Botkin et al., 2003) and prophylactic mastectomy (Schrag et al., 1997) Well-informed participants are likely to be similarly vigilant in the context of preclinical brain disorders.

Preclinical detection can offer the opportunity to plan for the predicted disorder, even if disease progression cannot be influenced. Patients and caregivers are often faced with an impending change in personality and behavior, demonstrating another unique realm for the treatment of preclinical brain disorders. The multidimensional contextualization of brain disorders often requires changes in the social environment, employment expectations, and independence. For example, an early diagnosis of ASD can allow a family to establish a home treatment plan or move the family to a location with strong support services (Sarrett and Rommelfanger, 2015). The definition of ASD (Pennington et al., 2014) and resources available for support services vary by locale, meaning relocating can substantially impact the child’s and family’s outcome. Similarly, awareness of developing schizophrenia or Alzheimer’s disease can initiate a caregiver relationship, giving the patient and provider more time to prepare and plan. Preplanning is crucial for caregivers, who often have to leave or transition their careers to care for their loved ones full time.

### Living with a preclinical diagnosis

If patients choose to be informed of a preclinical status, they face the risks of living with a preclinical brain disorder. Patients with psychosis anticipated that they would experience stigma in their interpersonal relationships and employment (Cechnicki et al., 2011), suggesting that a preclinical diagnosis could impact patients, even if the diagnosis is kept confidential. The fear of anticipated stigma could prevent patients from sharing their diagnosis, leading to social isolation and preventing predisease planning and the establishment of a caregiver. The knowledge of one’s status could also impair performance via stereotype threat. As an example, APOE4+ patients who were informed of their status performed worse on memory tests than those who were not informed (Lineweaver et al., 2014), and there is no evidence to suggest that reaction to a preclinical Alzheimer’s disease diagnosis would be any different. In the case of ASD, in which parents are the ones to receive their child’s diagnosis, parents may begin to treat a preclinical ASD child differently even before social deficits arise (if they ever arise). The change in family dynamics could be detrimental to the all family members, even those not diagnosed with the disorder.

If the patient chooses to disclose a preclinical status or is in a scenario without the ability to choose (e.g., the results are automatically placed on their medical record), they become vulnerable to structural stigma and discrimination. In the United States, patients with preclinical diagnoses are not protected under the Americans with Disabilities Act because they have no current diagnosed disability. If information on preclinical status is made accessible, the law would need to be changed to afford protections. The United States Genetic Information Non-Discrimination Act can serve as a model for protecting patients from discrimination of preclinical status (United States Equal Employment Opportunity Commission, 2008), but no such legislation currently exists for biomarkers (Arias and Karlawish, 2014). The lack of standards surrounding how to treat individuals with a preclinical diagnosis leaves scientists and clinicians with the obligation to contribute to policy decisions, lest the science of preclinical detection outpace its legal and political frameworks.

The prospects of living with a preclinical diagnosis must include emergent and future technologies. In reality, all people are patients in waiting; all people are in a preclinical state for something. It is not simply that up to 36% of people ages 85 and above live with Alzheimer’s disease (Alzheimer’s Association, 2019). As predictive biomarkers emerge and the technology to detect them improves, every asymptomatic person will qualify for some preclinical diagnosis. Therefore, research must understand and develop procedures on how to best live with a preclinical diagnosis in social, legal, and personal realms because those decisions will affect an increasingly large percentage of the population.

## Communicating Risk

Much of the burden to ensure ethical preclinical research and screening will fall on the teams conducting the work. Relative risk is poorly understood on a conceptual level, so the practical effects of a patient’s status must be described and discussed by the research/health care team. Teams directly involved in preclinical detection already recognize there is a difference between a statistically significant risk factor and a reason to change behavior. As an example, one team found that those in the top decile of risk profile scores (RPS) by genetic analysis had an odds ratio >7 of developing schizophrenia. Although this is statistically significant and a substantial effect, the authors acknowledge that this information would have little real-world utility for patients and recommend against using the RPS as a predictive tool (Schizophrenia Working Group of the Psychiatric Genomics Consortium, 2014). However, patients will have a right to know their status when similar tools are introduced into the clinical setting, which will require deliberate communication between patients and clinical providers. Many individuals, scientists included, could feel that being seven times more likely than the average person to develop schizophrenia makes the disorder inevitable, when in reality they would have approximately a 7% chance of developing it in their lifetime (World Health Organization, 2001). High relative risk could be easily interpreted as certainty, so information must be contextualized as part of a larger discussion about what a diagnosis could mean for the patient.

Before disclosure of preclinical diagnoses becomes common practice, an agreement of when to disclose must be established. The relative value and risks associated with Type II (false negative) and Type I (false positive) errors will be an inevitable part of preclinical detection, since biomarkers for developmental brain disorders are inherently probabilistic. In scenarios where health is not immediately compromised, high Type II error may be preferred over high Type I error, but these calculations would be different for every disorder and biomarker. The validity of that statement will only be determined by systematic research into public attitudes. An online survey by the Mayo Clinic (Caselli et al., 2014) found that the majority of respondents from an Alzheimer’s disease prevention registry would undergo biomarker testing if given the choice, and that the results of such testing would influence positive lifestyle changes. However, a significant minority reported that a high-risk status would prompt them to “seriously consider suicide.” This self-report is at odds with many reviews of health outcomes following the disclosure of risk status, which claim that the information tends to, at worst, induce transient anxiety or depression (Paulsen et al., 2013; Kim et al., 2015). In fact, patients were found to over-rate negative health outcomes and were more resilient than initially predicted. Even so, the extreme negative response of a subset of the population cannot be ignored. That, and the indeterminate effects of a preclinical diagnosis on stigma, employment, and health care highlight the need for risk disclosure to be integrated into psychological screening and counseling.

## Recommendations

Preclinical detection of brain disorders, both for research and clinical purposes, impacts patients in unique ways. The introduction of detection technologies will likely not be controlled by the scientific community. Other groups have already noted that preclinical tests may be integrated into diagnoses by market and consumer pressures rather than by scientific consensus (Racine et al., 2017). Therefore, the introduction of these technologies cannot be passively integrated. Rather, standards for preclinical research and diagnoses must be established in anticipation of their adoption. These standards should be cocreated with the input of diverse stakeholders, including patients, policy makers, scientists, and health care providers.

Even the practice of informed consent will need to be restructured in the context of preclinical detection. Longitudinal studies concerning brain disorders demand a custom consent protocol: a fully competent and autonomous patient at the beginning of a study may progress to a point of diminished capacity and autonomy over the course of the study. Standards of reconsenting a patient must be established and communicated to the patient (and applicable caregivers/powers of attorney) at time of enrollment.

Furthermore, the consent process must include all possible outcomes and results, not only those directly related to the brain disorder of primary interest. As the predictive power of preclinical biomarkers improves, more and more tests will have the potential to uncover incidental findings of a preclinical diagnosis. The search for biomarkers to diagnose a clinical disorder will likely include incidental and secondary findings, which, with the increasing availability of genetic testing, have already permeated clinical settings. Citing a duty to prevent harm to patients, the American College of Medical Genetics and Genomics (ACMG) recommended that all clinical genomic sequencing be coupled with tests for a predetermined list of pathogenic markers. More controversially, the ACMG recommends that the patient should not be given the opportunity to refuse either the test or the receipt of the results (Green et al., 2013). Their recommendation has caused many critics to cite a lack of respect for patient autonomy (Wolf et al., 2013), and other commissions have argued in favor for a patient’s right to refuse (Weiner, 2014). Although incidental findings from intensive screenings may be inevitable, the distress of such findings on patients is not. The consent process must inform the participant of known secondary findings and the possibility of incidental findings. The participants’ preferences to know or not know should be integrated into the consent process, and neither decision should be a criterion for exclusion from the study or trial. After all, the effects of incidental preclinical findings on a patient’s life will change once the findings become more reliable predictors of the development of a clinical state. Chneiweiss (2017) has argued that ethical use and disclosure of preclinical biomarkers is dependent on their use to the patient, and the utility of these markers are continually changing. Thus, guidelines for the primary or incidental detection of preclinical biomarkers must be regularly reevaluated to accurately reflect the relationship between patient and preclinical diagnosis. A positive model for such guidelines is the policy of the Wellcome Trust, which, without mandating a specific course of action by research groups, requires a concrete and well-justified policy on the disclosure of incidental findings as a condition for funding (Wellcome Trust, 2014).

In preparation for potentially disclosing results to a participant, clinicians and scientists would benefit from formal risk communication training. The qualities of effective communication cannot be assumed; the development of effective communication will require empirical research on how the public best understands and receives data on preclinical risk. In fact, the Presidential Commission for the Study of Bioethical Issues (Weiner, 2014) recommended that clinicians disclose absolute risk to patients instead of relative risk, although the genetic tests discussed by the Commission directly informs relative risk. Such reports suggest the most effective way to communicate relative risk is to translate it into a more intuitive metric. Here, partnerships with advocacy groups focused on specific diseases will be invaluable. Organizations such as Autism Speaks or the Alzheimer’s Association form relationships between all parties affected by the contextualized brain disorder, from the patients to the caregivers, to the physicians, to the politicians. In addition, the advocacy work of these organizations has fostered trust in the community, which will be crucial to reach historically underserved populations (Dawson and Bernier, 2013; Cahill et al., 2015).

Deliberate public engagement will also improve the impact of a preclinical diagnosis. Patients prescribed antipsychotic medications were more likely to stay on their medication schedule and had improved health outcomes when they engaged in integrated pharmaceutical and non-pharmaceutical interventions, such as through community health partners (Zygmunt et al., 2002). Psychoeducational and family therapy programs, although common, had poorer outcomes than behavioral programs or case management (Zygmunt et al., 2002), showing how intuitive interventions are not always the most effective. For brain disorders with no current treatment, multidimensional treatment approaches may be more effective than traditional pharmaceutical interventions. A multidomain intervention, which included diet, exercise, cognitive training, and vascular risk monitoring, prevented cognitive decline in elderly people at risk of developing Alzheimer’s disease to a greater extent than a basic health advice intervention (Ngandu et al., 2015). This landmark study should serve as a reminder that preclinical research should not be restricted to the development of pharmaceuticals. It is imperative to capitalize on integrated and objectively measured strategies. Doing so will not only maximize therapeutic potential but will also facilitate public cooperation and trust.

It must be acknowledged that a significant potential for harm to patients may arise from existing legal standards, or lack thereof. Protections and rights of patients must be formalized before official preclinical diagnoses are put into practice. Considerations should include what information can be shared with the patient’s health insurance provider and the patient’s employer, as well as what protections should be put in place to guard against discrimination in the workplace. As the ability to detect preclinical stages of disorders improves, standards must also contain protections against forced testing and disclosure of results. Given the loss of productivity associated with disorders like schizophrenia and Alzheimer’s disease (Takizawa et al., 2015; Chong et al., 2016), screening employees for such risk could be an economic advantage for the employer. Again, examples from how individuals are protected against maltreatment due to other biomarker status offer positive models. Nonsense mutations of monoamine oxidase A (MAOA) were one of the first genetic biomarkers associated with aggressive behavior and criminality (Brunner et al., 1993). Although the original team did not advocate the use of the MAOA marker to classify individuals as criminals or likely recidivists (Brunner, 1996), many worried that the MAOA biomarker would be used as a eugenic classification. Especially given the gene by environment interaction influencing the effect of MAOA status on behavior (Kim-Cohen et al., 2006), Baum and Savulescu (2013) argued ethical uses of MAOA status must focus on protection of the individual, not preemptive action taken against the individual. The same is true for individuals who carry a preclinical biomarker for a brain disorder; reactions to a preclinical diagnosis must focus on the mobilization of resources to prepare for the increased likelihood of a future clinical state. The use of biomarkers alone, be they preclinical biomarkers of Alzheimer’s disease or the MAOA allele, are not sufficient to fully predict future behavior. Additionally, biomarkers alone are not sufficient to justify a change in how an individual is employed, treated, or how autonomy or agency is recognized. Harm is inevitable if the scientific possibilities outpace the legal framework in which they reside. Therefore, it is incumbent on scientists involved in the research of preclinical detection of brain disorders to also be active advocates for patient-forward policy standards.

## Conclusion

Brain disorders are becoming statistically more prevalent in a population that is living longer and that is less affected by communicable diseases (Borlongan et al., 2013; Effertz and Mann, 2013). We must recognize that everyone is a patient in waiting. All disorders are developmental in nature, and therefore many more disorders than those discussed above have discrete, if currently undiscovered, preclinical stages. Risk modification will be the future of health care as the science of preclinical detection progresses. A thorough investigation of best ethical practices is needed to manage the use of new tools in the clinic and beyond. Regulatory hurdles and public distrust can easily stymie or corrupt these advancements if scientists and clinicians fail to engage in conversations with policymakers and the wider public. Most importantly, we must recognize that the best practices will not be consistent across conditions or cultures. True appreciation for the risks of preclinical research requires the acknowledgment that the risks (be they stigma, impact on interpersonal relationships, or individual anxiety) are influenced by cultural norms. The need for empirical research to measure public attitudes is never more important than when identity and autonomy are directly impacted. We can maximize scientific advances and public acceptance by responding to, and not dictating, public views on the matter. Such a dialogue will help the scientific community protect patients before the harms of uninformed preclinical detection are inflicted on them.

## Synthesis

Reviewing Editor: Rae Silver, Columbia University Decisions are customarily a result of the Reviewing Editor and the peer reviewers coming together and discussing their recommendations until a consensus is reached. When revisions are invited, a fact-based synthesis statement explaining their decision and outlining what is needed to prepare a revision will be listed below. The following reviewer(s) agreed to reveal their identity: Marie Gibson, Adrian Carter.

Overview: This manuscript raises important ethical issues as there is constant progress in developing preclinical biomarkers for neural diseases. The manuscript is a potentially important contribution to the readership of eNeuro, as it covers ethical challenges that are becoming more pressing as the development of preclinical biomarkers of neural diseases advances. While the manuscript raises interesting and important points, it is not clearly directed at its presumed target audience. The issues facing clinicians and researchers differ- and these should be clarified. The status of biomarkers for “mental illness” or “brain disease” as an immature science is not sufficiently clearly stated. There is a significant body of literature relevant to the present review- this can be cited more thoroughly.

The ethics of preclinical testing for neurological and mental disorders has been covered elsewhere in the literature, including in scientific and medical journals, and in the ethical literature, but these sources are inadequately referenced. The value of the current paper lies in its potential to focus on aspects that are of specific interest and working scope of the eNeuro and Society for Neuroscience community. This is an important topic that should be covered in journals such as eNeuro, but contributions of this manuscript in the context of the field as a whole can be strengthened.

As noted, there are risks with prematurely treating a condition based on very early diagnosis. There are also important ideas as to the responsibility of scientists and clinicians for advocacy relevant to patient privacy and autonomy such as urging clinicians and scientists to advocate for patient autonomy (including the right to refuse treatment) and privacy. Using three “life stages” of possible neural deficits provided interesting contrasts. Also considered are the importance of comprehensive consent forms.

The focus on disorders that may occur at three stages of the life cycle provides insight into how each stage presents some specific issues. The risk of prematurely diagnosing autism spectrum disorder {ASD} vs. doing the same for schizophrenia provides an interesting contrast. For example, the proposed behavioral interventions for possible ASD are beneficial for normal children as well, which supports their beneficence, whereas some of the antipsychotics that may be prescribed for possible schizophrenics may have severe side effects. On the other hand, preclinical biomarkers are already available for Alzheimer's disease, and their use may provide an opportunity for treatments to be more effective but may also prematurely lead to violations of patient privacy. The issues raised are widely relevant to other neural disorders.

It is wise to emphasize the need for clinicians and scientists to advocate for patient autonomy and privacy. The author(s) recommend measures to address issues of patient autonomy and privacy. This manuscript points out that informed consent in clinical trials must emphasize the limited or absent personal benefit that is likely for the patient. Clinicians and scientists should be trained to more effectively communicate the meaning of “risk” in a more understandable way. They also need to be involved in policy decisions to avoid discrimination against those with preclinical diagnoses. The standard of doing no harm is closely intertwined with patient autonomy and privacy.

There is a valuable discussion of the consent process. The principle of patient autonomy should ensure that the patient has the right to refuse a test, as well as the right to refuse to learn the result of a test.

The authors examine the ethical issues raised by research and potential clinical use of various technologies to predict mental and neurological disease, prior to their onset. These developments raise important ethical, social and legal issues that need to be considered by researchers, clinicians and policy makers. However, there are significant weaknesses in the paper in terms of material covered and its presentation that need to be addressed. Specifically, scholars in the field of neuroethics have been examining this since the field's inception in ~2002. Many of the same issues have been considered in other areas of medicine by bioethicists. The current paper needs to build more throroughly on this previous work to show how what it adds to the field is novel.

The authors' claim that “preclinical detection of brain disorders encompasses a unique suite of ethical concerns” can be clarified. Many of the ethical concerns raised are also pertinent to clinical research in general (e.g. informed consent, respect for persons, beneficence). How these ethical concerns play out in the case of preclinical testing of mental illness needs attention.

The science behind predicting brain diseases is not new, but has not yet been phenomenally successful. While the ethical considerations are significant, the current challenge is to find robust neurobiological markers that can predict mental illness remains, despite significant advances in understanding the genome and epigenetic factors. For behavioral disorders, current technologies are unable to predict disease with sufficient specificity to be useful, and may be the result of the complex biological, psychological and social factors involved. This point is not clearly made.

Key concepts in the paper should be more clearly defined. What is a “brain disease” for instance? Just because there are differences in the brains between large groups of people with a mental illness and those without, does not make it a disease. Need to more clearly define what illnesses they are discussing. The ethical issues raised between neurological disorders of acute organic origin are very different to complex biopsychosocial mental illnesses like depression and addiction.

The scope of the technologies being discussed is also not clear - mention eye tracking through to PET scans. In particular, it is not clear whether the authors are primarily concerned with the use of these technologies in research or in the clinic. Applications in these contexts raise very different ethical considerations and responses. If there is no evidence to support their clinical use, arguably the paper might focus more squarely on the research ethics, and provide more specific and usable guidelines. This is most obvious in section 4 - the communication of findings are very different in research and clinical settings (where presumably the evidence of predictive accuracy and the availability of treatments supports its clinical use; whereas research settings aim to develop that evidence). This is a key issue that is not covered in the paper. The authors' discussion of the clinical use of these technologies is often contradictory (see p.5-6).

In discussing the communication of findings to patients/participants, the authors do not consider who it is that communicates any clinically relevant information. Ideally this should be done by a trained counsellor (as is done in genetic research). Participants in research should also be informed at consent whether they will be told about any results of their participation, as well as how incidental findings will be dealt with. There are many other issues that have been covered in the literature that have been given insufficient treatment in this paper. The coverage of the ethical issues raised is rather superficial in comparison. The paper describes three different conditions in which preclinical testing could be applied. This provides an opportunity to tease out how the ethical issues apply in these different contexts and highlight the issues that need to be balanced. However, this is not done in the discussion.

There are also a number of important ethical issues that have not been discussed: the issue of limited predictive accuracy, potential for medicalisation of complex behavioral disorders, the impact of predictive testing on people undergoing unnecessary medical procedures, dealing with incidental findings (or what is an incidental finding in a research context), and the potential impact on people's self-understanding and health-related behaviur.

Finally, the paper will benefit from careful editing, removing repetitive sections and grammatical errors, and referencing.

The reviewers suggest consultation of the following key neuroethics textbooks in the area that feature good review chapters of the issue:

Illes, J. ed. 2017. Neuroethics: Anticipating the future. Oxford: Oxford University Press.

Clausen, J., and N. Levy. 2015. Handbook of neuroethics. Springer.

There is also an entire book devoted to the neuroethics issues that includes a number of relevant chapters:

Singh, I., W. P. Sinnott-Armstrong, and J. Savulecu. 2013. Bioprediction, biomarkers, and bad behavior: Scientific, legal, and ethical challenges. Oxford University Press.
